# Size, longevity and cancer: age structure

**DOI:** 10.1098/rspb.2016.1510

**Published:** 2016-09-14

**Authors:** Maarten J. Wensink

**Affiliations:** 1 Max Planck Odense Center on the Biodemography of Aging, University of Southern Denmark, Winsløws Vej 9B, 5000 C Odense, Denmark; 2 Institute of Public Health, University of Southern Denmark, Winsløws Vej 9B, 5000 C Odense, Denmark

**Keywords:** multicellularity, evolution, Peto's paradox, cancer, longevity

## Abstract

There is significant recent interest in Peto's paradox and the related problem of the evolution of large, long-lived organisms in terms of cancer robustness. Peto's paradox refers to the expectation that large, long-lived organisms have a higher lifetime cancer risk, which is not the case: a paradox. This paradox, however, is circular: large, long-lived organisms are large and long-lived *because* they are cancer robust. Lifetime risk, meanwhile, depends on the age distributions of both cancer and competing risks: if cancer strikes before competing risks, then lifetime risk is high; if not, not. Because no set of competing risks is generally prevalent, it is instructive to temporarily dispose of competing risks and investigate the pure age dynamics of cancer under the multistage model of carcinogenesis. In addition to augmenting earlier results, I show that in terms of cancer-free lifespan large organisms reap greater benefits from an increase in cellular cancer robustness than smaller organisms. Conversely, a higher cellular cancer robustness renders cancer-free lifespan more resilient to an increase in size. This interaction may be an important driver of the evolution of large, cancer-robust organisms.

## 1. Introduction

Multicellularity is risky. Every cell could, in principle, escape the checks and balances of healthy organisms that keep individual cells from proliferating in an uncontrolled manner and cause cancer [1–3]. To do so, a cell needs to differ in a number of ways from normal cells (i.e. rate limiting stages or ‘hits’). This observation has given rise to the ‘multiple hit model’ or the ‘multistage theory of cancer’ [4–8]. Every ‘hit’ is a way in which cancer cells necessarily differ from normal cells. For instance, a cancer cell needs to sidestep the checkpoints in the cell cycle. Most hits seem to result from DNA mutations, whereas epigenetic mutations may also play a role [9–11]. For brevity, I write ‘mutations’ and ‘stages’.

It has long been recognized that with *c* stages, the cancer hazard rate should rise approximately as a power of *c* − 1 with age, and many cancers seem to have a hazard function that is at least approximately compatible with this model [4].

If having many cells is risky, then having even more cells should be even riskier. If the hazard rate increases with age as a power of *c* − 1, then a longer life should progressively increase cancer risk. Hence, large, long-lived organisms are expected to suffer a higher lifetime cancer risk than small, short-lived organisms. This does not seem to be the case; an apparent contradiction known as Peto's paradox [12,13] that is receiving increasing attention from the medical community [14,15].

Peto's paradox [15–21], however, is circular. The paradox relies on assuming a certain lifespan, after which the cancer risk during that lifetime is evaluated. This seems the wrong procedure. Lifespan is a function, inter alia, of cancer robustness: organisms are long-lived *because* they are cancer robust. If not, then they would be short-lived. One cannot next expect that they are not cancer robust and should therefore have a higher lifetime cancer risk, based on the very same lifespan that derives from high cancer robustness. Similarly, large organisms exist because they are cancer robust; one cannot next expect that they are not.

Formulations like the following are equally uncomfortable: ‘the risk of cancer should be many orders of magnitude greater in humans [than in mice]’ [17]. Lifetime cancer risk in mice is at least one-third [22,23], so lifetime cancer risk in humans cannot possibly be orders of magnitude higher.

**Figure 1.**
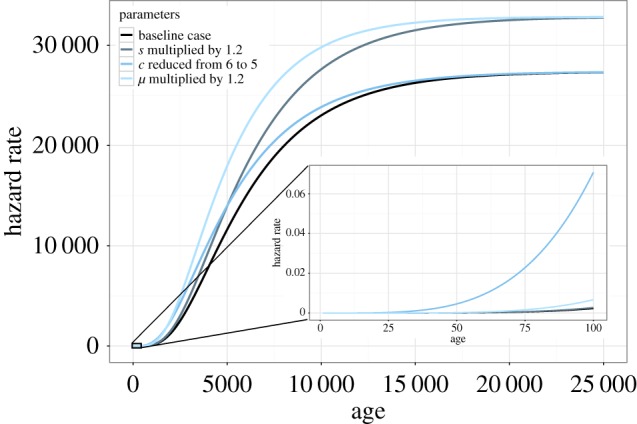
The hazard rate as a function of age for several parameter settings. The black line is the reference model. The number of mutations that gives cancer, *c*, changes the shape of the hazard rate (dark blue line). The number of cells at risk, *s*, scales the hazard rate (grey line). Mutation rate *μ* gives the accelerated failure time effect (light blue line). Note that the plateau is not reached during normal lifespan. (Online version in colour.)

To give another example, Peto mentions the rapid increase with age of cancer risk up to that age, the implication being that a longer life leads to a progressively higher lifetime cancer risk [12]. Apart from the objection to this procedure raised above, a *steeper* increase of the cancer incidence rate (and risk) with age actually *reduces* cancer risk up to any specified age, cancer being postponed to later ages (figure 1).

Clearly, the conceptualization of these matters in terms of lifetime risk invites unsound reasoning. In addition, lifetime risk does not reveal whether organisms die at age 1, or at age 100. Without other causes of death, ‘competing risks’ in the epidemiological literature [24–26], lifetime cancer risk is 1. With competing risks, lifetime cancer risk depends entirely on the way age distributions of cancer and competing risks interact. Hence, a lifetime risk is always situational: it is true only in the context of a specific set of competing risks.

A recent paper has investigated lifespan extension as a result of a change in cancer dynamics, exploring a theoretical model of cancer in the presence of a specific competing risk in the form of constant ‘extrinsic mortality’ [27]. Thus, organisms die of either cancer or ‘extrinsic mortality’, whichever strikes first, and overall lifespan is calculated. This approach overcomes the problems related to lifetime cancer risk, but the reported results hold only under a constant competing risk hazard. For instance, a constant ‘extrinsic mortality’ rate of 0.1 per year implies that overall lifespan cannot be extended beyond 1/0.1 = 10 years, regardless of cancer dynamics. But there are various non-cancer mortality functions other than a constant mortality rate of 0.1 that limit lifespan at 10, for instance the Gompertz function *ae^bx^* with *a* = 0.001 and *b* = 0.579701. The perturbation that Kokko & Hochberg [27] report to result in a lifespan reduction from 9.40 to 7.79 (their figure 1*d*) then instead yields a lifespan reduction from 9.99 to 9.74: a significantly different result.

Because there is no generally prevalent set of competing risks, it is instructive to temporarily dispose of competing risks and investigate the pure age dynamics of cancer: cancer-free lifespan, its coefficient of variation and its sensitivity to model parameters. Here, I show how such a theory could take shape, how earlier results can be augmented and how new exciting results can be obtained along these lines. I analyse a straightforward model of cancer age incidence under the multistage model of carcinogenesis that is a slightly adapted version of the Calabrese–Shibata model [28] also analysed in various recent papers [16,27,29]. Yet it can be analysed even deeper, with surprising results: in terms of cancer-free lifespan, large organisms reap greater benefits from an increase in cellular cancer robustness than smaller organisms. Reversely, a higher cellular cancer robustness renders cancer-free lifespan more resilient to an increase in size. This interaction may be an important driver of the evolution of large, cancer-robust organisms.

The model [28] is the most direct derivation of cancer age incidence under the multistage model of carcinogenesis, making it fundamental to cancer research. Achieving a good understanding of the model dynamics is therefore of considerable interest. It should be emphasized, however, that various biological factors that influence carcinogenesis are not in the model, such as clonal expansion, selection and varying mutation rates, making the predictions inexact at best. Nevertheless, the model does chart the basic machinations of a process that is widely believed to be fundamental to carcinogenesis, and it serves well to highlight important theoretical aspects.

## 2. Model analysis

Suppose that cancer requires *c* mutations, stages. Suppose that an organism consists of *s* potentially malignant cells. Further suppose that genes mutate at a per time rate *μ*. Let *Z* denote the time to mutation of an individual gene. The probability that *Z* exceeds *x*, so that the gene is not mutated at age *x*, is then2.1
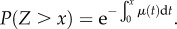
With constant *μ*, this gives2.2
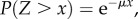
but the original equation may be used for more involved modelling [4].

Let *Y* denote the healthy survival time of an individual cell, which ends if *c* mutations have occurred;2.3
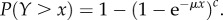


Let *X* denote the healthy survival time of an entire organism. For an organism to be cancer-free, all cells need to be cancer-free. For *s* cells, the cancer-free survivorship up to age *x* for the entire organism is2.4
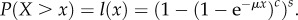
Equation (2.4) is the same as the equation originally derived by Calabrese & Shibata [28], only now in continuous time rather than in ‘cell division time’ and for an entire organism rather than for the bowel alone. The same equation (or similar) is found in [16,27,29].

To explore age patterns, probability density function *f*(*x*) is calculated as2.5
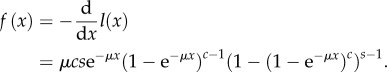
Change in survivorship *f*(*x*) comes down on those organisms still alive (i.e. cancer-free), expressed by the *hazard rate λ*(*x*),2.6
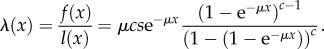
The total number of stem cells in humans seems to be in the order of 10^11^ [10], but organisms like elephants and whales are clearly expected to have many more. I follow earlier work [16,27] in taking a yearly mutation rate of *μ* = 0.00027375. The number of mutations necessary for cancer may be as low as two [7] or three [30], but is thought to be typically higher, in the range of 3–8. I explore a wide range of parameter values as appropriate.

It was recognized long ago [4] that with *c* stages the hazard rate should increase by a power of *c* − 1 with age. However, this is true only initially. The population increasingly consists of organisms of *s* cells waiting for their last mutation (all other organisms already have cancer), which comes at mutation rate *μ*, meaning that the hazard rate limits at *sμ*. This explains why the hazard rate increases faster with age in relative terms for larger *c* (initially increasing as a power of *c* − 1 with age), whereas one would expect, *c* being a cancer-robustness mechanism, that the hazard rate would be higher for smaller *c* than for larger *c* at all ages. Through the limit at *μs*, such is, indeed, the case (figure 1).

The hazard rate is helpful in charting the effects of parameter changes (figure 1 and equation (2.6)). Multiplication of *s* by some factor *ϕ* > 0 multiplies the hazard rate by *ϕ* for all ages: *s* scales the hazard rate, which means it gives a proportional hazards model [31]. A change in *c* does not change the level of the plateau, but changes the way the curve approaches the plateau. If *c* is higher, the hazard rate stays lower for longer, but eventually catches up. Finally, *μ* not only scales the hazard rate, but also its time dimension: multiplying *μ* by some *ϕ* > 0 changes *λ*(*x*) to *ϕλ*(*ϕx*) and *f*(*x*) to *ϕf*(*ϕx*) (equations (2.6) and (2.5)), whereas the same survivorship would be reached at *x*/*ϕ* (equation (2.4)). This model is known as the accelerated failure time model [31], which means that a straightforward relationship exists between *μ* and survivorship: the distance between any two points is multiplied by 1/*ϕ* in the age dimension, but except for this scaling the survivorship function is identical.

**Figure 2.**
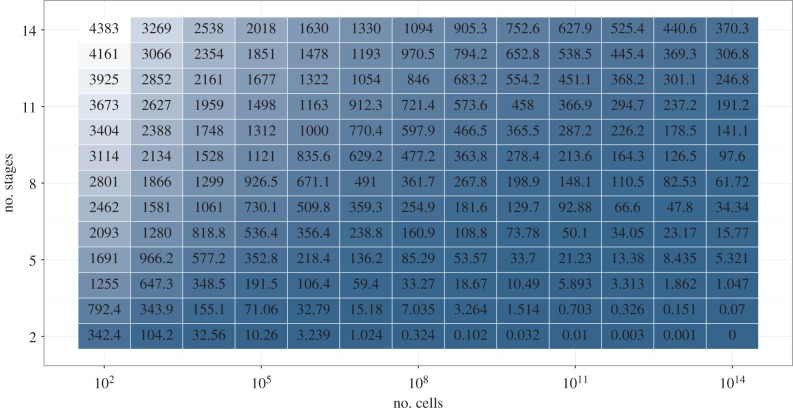
Cancer-free lifespan for several parameter settings. Mutation rate *μ* is fixed at *μ* = 0.00027375, because the effect of *μ* is described entirely by the accelerated failure time model. The number of stages, *c*, and the number of cells at risk, *s*, seem to interact. Compare for instance the change from *c* = 6 to *c* = 8 for *s* = 100 increasing 10-fold to *s* = 1000 versus the same change in *c* for *s* = 10^10^ increasing 10-fold to *s* = 10^11^. In the first case, the original lifespan is not recovered. In the second case, the original cancer-free lifespan is more than doubled. Note that the step size for *s* is multiplicative, while being additive for *c*.

Cancer-free lifespan, calculated as the first moment around 0 of *f*(*x*), or as the sum under the survivorship curve [31], is key in any framework of analysis. The accelerated failure time property of *μ* means that multiplication of *μ* by *ϕ* amounts to multiplying cancer-free lifespan by 1/*ϕ*, with no surprising effects. For parameters *c* and *s*, effects are shown in figure 2: organisms that are orders of magnitude larger (high *s*) need only a slightly higher number of stages (higher *c*) to achieve the same cancer-free lifespan. In addition, figure 2 suggests that this effect is stronger the larger *s* is.

**Figure 3.**
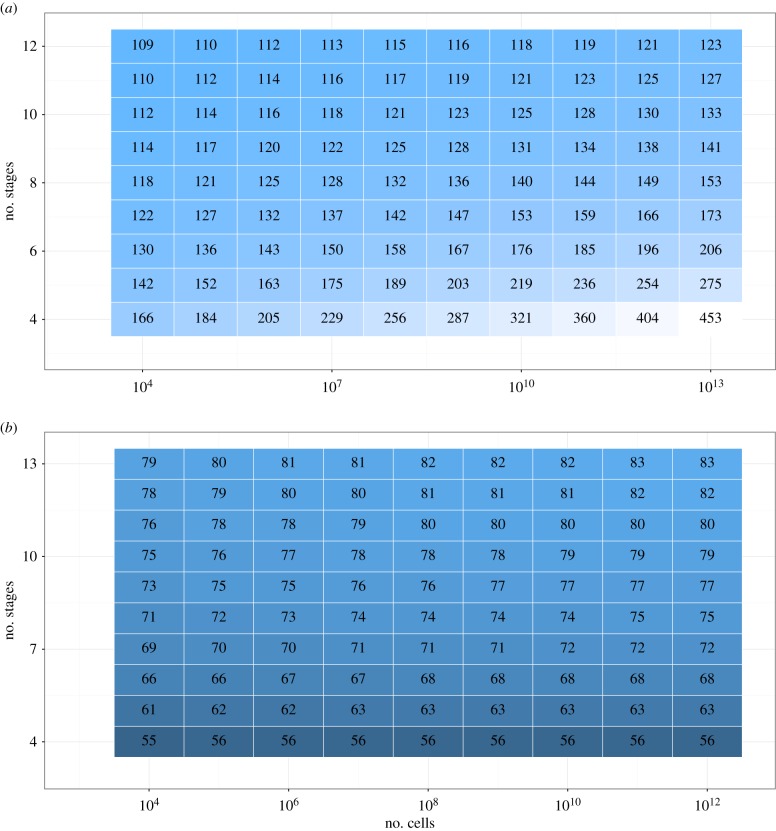
Cancer-free lifespan as a percentage of old lifespan (*a*) following a step increase in *c* while keeping *s* constant, and (*b*) following a step increase in *s* while keeping *c* constant. For example, for an organism characterized *s* = 10^6^, *c* = 5, an increase in *c* from 5 to 6 would result in an increase in cancer-free lifespan by a factor of 1.63, whereas an increase in *s* from 10^6^ to 10^7^ would result in a decrease in cancer-free lifespan by a factor of 0.62 (*μ* = 0.00027375).

The interaction between *c* and *s* is further explored in figure 3. Figure 3*a* shows a heatmap of cancer-free lifespan after an increase in *c* as a percentage of cancer-free lifespan before that increase while keeping *s* constant. Figure 3*b* shows a heat map of cancer-free lifespan after an increase in *s* as a percentage of cancer-free lifespan before that increase while keeping *c* constant. For an increase in *c*, these percentages are greater than 100, because increasing *c* increases cancer-free lifespan. For an increase in *s*, these percentages are smaller than 100, because increasing *s* decreases cancer-free lifespan. Significantly, the effect on cancer-free lifespan following an increase in *s* is smaller when *c* is larger, whereas the effect on cancer-free lifespan following an increase in *c* is larger when *s* is larger. Hence, the larger the organism, the more it gains from an increase in *c*, whereas the higher *c*, the smaller the proportional reduction in cancer-free lifespan following an increase in *s*. A similar interaction between *c* and *s* occurs for their effectiveness in reducing the coefficient of variation (electronic supplementary material, figure S1).

## 3. Discussion

The findings in this paper demonstrate the benefits of cancer-free survivorship rather than lifetime cancer risk as the metric of interest in investigations regarding the evolution of cancer. This perspective was used to rethink Peto's paradox, which was found to be circular. Large, long-lived animals can exist if and only if they are cancer robust; one cannot next expect them to have a higher lifetime cancer risk because they are not cancer robust. The observation that (cells of) large, long-lived organisms must be more cancer robust than (those of) small, short-lived organisms is shrewd and of great importance, but should have been the endpoint. The expectation that large, long-lived animals should have a higher lifetime cancer risk than small, short-lived organisms is an unnecessary and faulty extra step, as is the resulting paradox when that prediction remains unconfirmed. Given that whales live up to 200 years and weigh up to 200 000 kg [32], their cancer dynamics differ from those of humans, and the ‘promise of comparative oncology’ [17] stands.

The relevance of the age distribution of cancer and competing risks has not gone unnoted. Various authors have noted that postponing cancer until after reproduction renders natural selection largely powerless in cancer suppression [12,29,33,34]. Lichtenstein [35] commented on the timing of cancer from the perspective of Peto's paradox: ‘animals with a small body weight and short lifespan (e.g. rodents) should not suffer from cancer at all, while big animals (whales) should get cancer in their mothers' wombs’. Noble *et al*. [18] mention both age distributions and competing risk in their reanalysis of Tomasetti & Vogelstein [10], as do Caulin *et al*. [16]. In several life-history models of cancer, competing risks (and hence necessarily age distributions) feature prominently [20,27,29]. Yet these papers have stopped short of disposing of Peto's paradox and placing cancer-free survivorship (rather than lifetime risk) at the heart of the theory, whereas the use of a specific set of competing risks limits the generality of the results. Lifetime risk or overall lifespan can certainly be calculated if one is interested in a specific set of competing risks, but keeping in mind that the results are restricted to situations where that set of competing risks applies.

A greater number of stages has been suggested before as a possible mechanism by which larger organisms can protect themselves against cancer [16,21,34,36]. Caulin *et al*. [16] found that ‘increasing the number of hits required for cancer was a powerful tumour suppressive mechanism’. This result presaged the findings here (figure 2), with the reservations that the effect of *c* depends on *s*, and that Caulin *et al*. look at cancer risk before age 90, subject to all the objections raised above, rather than cancer-free survivorship.

Caulin *et al*. [16] when exploring lifetime cancer risk in the Calabrese–Shibata model found that a 3.2-fold reduction in the mutation rate compensates for a 1000-fold increase in body size. This claim is at odds with the findings of others [27,29], who find that a doubling of *μ* halves cancer-free survivorship up to any point. The identification of the effect of *μ* with the accelerated failure time model confirms the results of [27] and [29], whereas the finding of Caulin *et al*. [16] seems an artefact of their parameter settings (electronic supplementary material, figure S2).

Kokko & Hochberg [27] call for mathematical models that explore how *c* and *s* co-evolve. They find that larger organisms gain more from an increase in *c* from 3 to 4 than smaller organisms (their figure 2*c*). They do not, however, show the dependence of the effect of a change in *s* on *c*, do not discuss the coevolution of *c* and *s*, and as noted their results depend on the specific, non-general set of competing risks that they consider, to wit age-invariant ‘extrinsic mortality’. Kokko & Hochberg [27] further write that reducing *μ* (their parameter *k*) has an effect similar to increasing *c* (their parameter *n*). The above-mentioned analysis shows that *μ* gives the accelerated failure time model, with no interactions with other model parameters, whereas the effect of *c* is not that of the accelerated failure time model, depending instead on *s* and on *c* itself. While both *μ* and *c* could be manipulated to postpone cancer, these manipulations work out differently.

Finally, Brown *et al*. [20] make the ‘assumption of diminishing returns to increased cancer suppression’, which is corroborated by the finding in this paper that a further increase in *c* becomes less beneficial the larger *c* is (but becomes more beneficial again following an increase in *s*).

The favourable interaction between *c* and *s* may be of paramount evolutionary importance: organisms may differ orders of magnitude in body size, but to equalize their cancer-free lifespans requires only a small number of additional cancer robustness mechanisms in the larger organism. This is effective *especially if organisms are large*. It would be interesting if experts in cellular biology could comment on how they view the costs of reducing *μ* versus increasing *c* in organisms of different sizes. Because extra robustness mechanisms (high *c*) are more effective in larger organisms (high *s*), larger organisms could let mutations run relatively free (high *μ*) as long as they assure some extra robustness in terms of high *c*. How does this weigh against the conflicting but equally reasonable hypothesis that mutation rates in larger organisms must be lower to protect them against cancer [21]? In addition, the reduction in variability as measured by the coefficient of variation could have evolutionary advantages, as it brings predictability to the life cycle. If the same life expectancy is reached through two different combinations of *c* and *s*, say (*c*_1_, *s*_1_) and (*c*_2_, *s*_2_) with *c*_1_ > *c*_2_ and *s*_1_ > *s*_2_, then (*c*_1_, *s*_1_) will have a more predictable life cycle than (*c*_2_, *s*_2_). For these reasons, mutually reinforcing effects of *c* and *s* uncovered here could be a major driver of the evolution of large, cancer-robust organisms.

A model is useful when it approximates reality. The model analysed here forms a good approximation of any cancer formation process that requires multiple discrete stages that are acquired at approximately constant rates over age. This model has been criticized [37], and the mathematical model explored here leaves out important biological factors that affect oncogenesis, such as clonal expansion, ageing, selection and varying mutation rates. There exist several ways in which the model could be made more involved. Non-stem cells could be modelled to have more stages than stem cells, as they are more phenotypically different from cancer cells, which could be modelled as subpopulations of cells with different *c*, *c* being higher for non-stem cells. Furthermore, mutations could increase the mutation rate itself, giving rise to the ‘mutator phenotype’ [38]. Eventually, tumours become complex adaptive systems, made up of a heterogeneous population of cells that compete, interact with their environment and undergo evolution by natural selection [9,33,39]. Nevertheless, it remains true that a number of modifications is required before cells become malignant [40] and the multistage model remains of interest [9,41].

Restricted to the basic logic of the multistage model of carcinogenesis, equations (2.2)–(2.4) describe not just *a* simple model; they describe *the simplest model possible*. Equation (2.2) is the general expression of survivorship as a function of a constant mortality rate (in this case, a gene remaining unmutated as a function of a constant mutation rate). Equations (2.3) and (2.4) then result from basic probability theory, and the density function and rate immediately follow from standard survival analysis. Therefore, for all its flaws and inaccuracies, the model analysed here represents the simplest case and serves well to demonstrate the theoretical points addressed in this paper. Perhaps more involved, more realistic models could build on these insights.

## Supplementary Material

Supplementary Figures S1 and S2
