# Supplementary material for: Size, longevity and cancer: age structure
Source: Proc Biol Sci. 2016 Sep 14;283(1838):20161510. doi: 10.1098/rspb.2016.1510 (PMC5031666; doi:10.1098/rspb.2016.1510)
Supplement: Supplementary Figures S1 and S2 [file rspb20161510supp1.pdf]

Supplementary Material to: *Size, Longevity and Cancer: Age Structure*

Maarten J. Wensink
Max Planck Odense Center on the Biodemography of Aging
Institute of Public Health, University of Southern Denmark
Winsløws Vej 9B, 5000 C Odense, Denmark
Email: mwensink@health.sdu.dk
Tel. +45 6550 9480
Fax +45 6550 3682

1 Supplementary figures

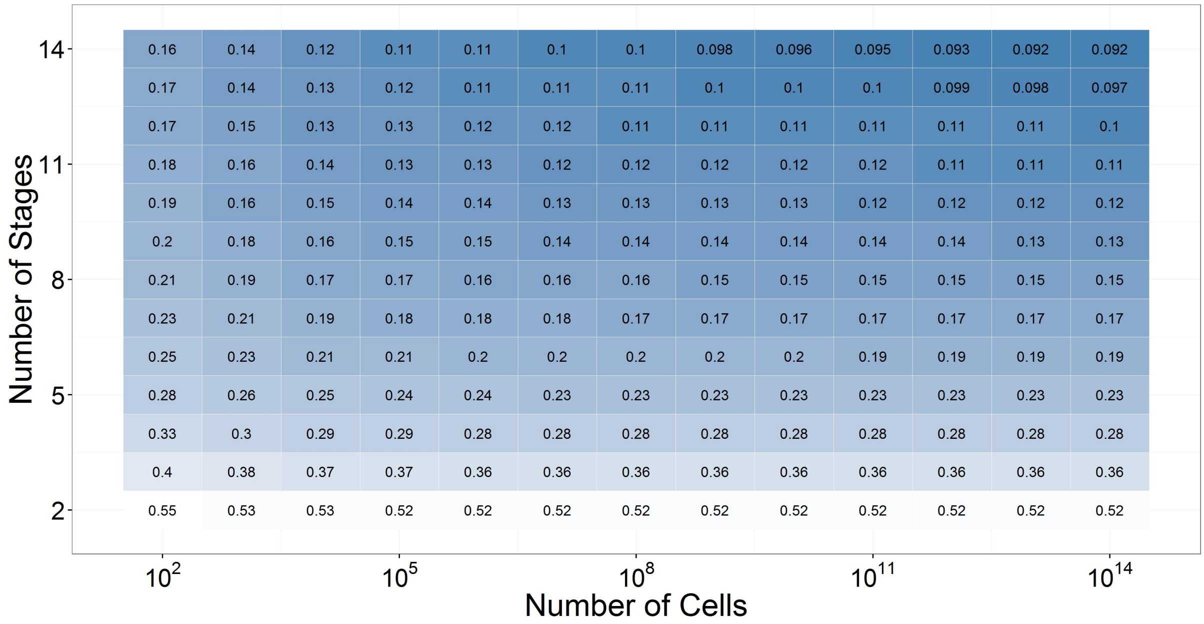

Figure S1: Heat-map of coefficients of variation for various parameter settings. For small c, s has virtually no effect on the coefficient of variation, following the observation that the model approaches the exponential distribution as c gets smaller. For $c = 1$, $l(x)|_{c=1} = (1-(1-e^{-\mu x}))^s = e^{-\mu s x}$, i.e. the exponential distribution with rate $\lambda = \mu s$, implying that the coefficient of variation is 1 and thus equal for all s (and μ) and that hazard rate λ is at the plateau for all ages. An increase in c reduces the coefficient of variation and again an intriguing interaction between parameters c and s is seen: if $c > 1$, an increase in s reduces the coefficient of variation and the more so the greater c is. Reversely, the same is true. Thus, c and s reinforce each other in reducing the coefficient of variation, which may have the important evolutionary advantage of increasing predictability.

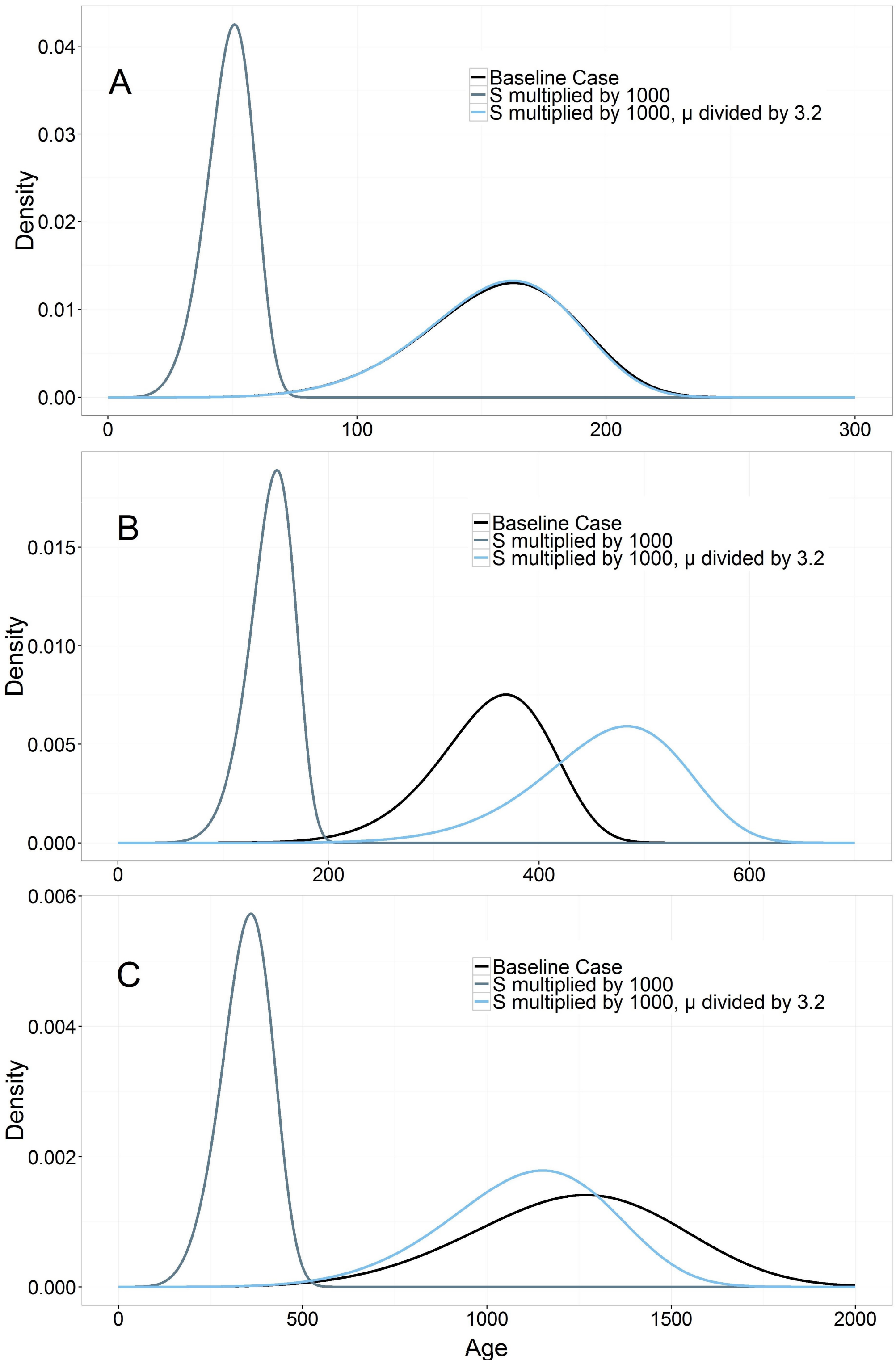

Figure S2: Density functions for various parameter settings. For the parameters used by Caulin et al. (reference 16 of the main text), a 3.2-fold reduction in μ does indeed approximately compensate for a 1000-fold increase in s (a). However, for other parameter settings this is not true (b and c), and the result of Caulin et al. seems an artifact of their parameter settings.
